# Over-expression of CDX2 alleviates breast cancer by up-regulating microRNA let-7b and inhibiting COL11A1 expression

**DOI:** 10.1186/s12935-019-1066-9

**Published:** 2020-01-10

**Authors:** Hongbin Wang, Yanlv Ren, Cheng Qian, Jiaxin Liu, Ge Li, Zhigao Li

**Affiliations:** Department of Breast Surgery (No. 2 Sickroom), Harbin Medical University Cancer Hospital, No. 150, Haping Road, Nangang District, Harbin, 150081 Heilongjiang People’s Republic of China

**Keywords:** Breast cancer, Migration, Invasion, CDX2, let-7b, COL11A1

## Abstract

**Background:**

microRNA Let-7 serves as a tumor suppressor by targeting various oncogenic pathways in cancer cells. However, the underlying mechanism of its involvement in breast cancer remains largely unknown. With our research, our endeavor is to explore the role of the CDX2/let-7b/COL11A1 axis in breast cancer cell activities.

**Methods:**

Tumor tissues and adjacent normal tissues were collected from 86 patients with breast cancer. Human breast cancer epithelial cell line MCF-7 was treated with over-expressed CDX2, let-7b mimic, shRNA against COL11A1 and their negative controls. The expression of CDX2, let-7b, and COL11A1 in the tissues and cells was determined by RT-qPCR. Interactions among CDX2, let-7b, and COL11A1 were detected by ChIP and dual-luciferase reporter assay, respectively. After different transfections, cell invasion, migration, and proliferation abilities were determined by Transwell and EdU assays. Lastly, tumor xenografts in nude mice were established and hematoxylin and eosin staining was performed to assess the tumor growth and lymph node metastasis.

**Results:**

CDX2 and let-7b were poorly expressed in breast cancer cells and tissues. CDX2 bound to let-7b and promoted the expression of let-7b, which contrarily inhibited the expression of COL11A1. Cancer cell proliferation, invasion, migration, and metastasis were stimulated when CDX2 and let-7b were depleted or COL11A1 was over-expressed. Xenograft tumors growth and metastasis were in accordance with the results of cellular experiments.

**Conclusion:**

In agreement with these observations, we could reach a conclusion that CDX2 could promote let-7b expression, which may exert an inhibitory effect on the proliferation, migration, and metastasis of breast cancer cells via repressing the expression of COL11A1, providing a novel therapeutic strategy for the treatment of metastatic breast cancer.

## Background

Breast cancer is the most common cancer in women, accounting for more than 30% of new cancer diagnoses in women [1]. Metastasis of breast cancer is a complicated process involving invasion and migration of cancer cells, and blood transversing, and it often associated with high mortality rates [2]. Unfortunately, therapies for metastatic breast cancer are limited, and remain to generally incurable [3]. Interestingly, a prior study suggested that breast cancer was affected by gene regulatory networks, in which transcriptional factors and microRNAs (miRNAs) were the most frequently mentioned. Hence, further exploration of the transcriptional factors and miRNAs in the progression of breast cancer might shed some light on prospective diagnoses and management therapies for the highly prevalent malignancy [4].

Transcriptional factors are well-known to play critical roles in numerous human diseases including cancers, and regarded as key regulators for new therapies [5]. One such factor, caudal type homeobox 2 (CDX2), a member of the caudal-related homeobox gene family and a mediator of apoptosis in tissues, was found in intestinal epithelial cells [6]. Recently, a study documented reduced CDX2 expression in breast cancer, while its methylation level is elevated [7]. Transcriptional factors have also been demonstrated to play important roles in breast cancer by interacting with miRNAs [8]. MiRNAs, also known as non-coding RNAs have been linked to mediate target genes expression and participate in different cancer cells proliferation, invasion, migration, and metastasis via several signaling pathways [2]. The let-7b was frequently reported with a decreased expression in cancers. For instance, the let-7 family members (let-7a, let-7b, and let-7g) have been demonstrated to be poorly-expressed in the patients diagnosed with breast cancer with lymph node metastasis [9]. Previous work further illustrated the targeting relationship between miRNA and collagen in breast cancer [10]. Interestingly, through bioinformatics and a dual luciferase reporter gene assay, COL3A1 was suggested as a direct target of let-7b in neuronal injury [11]. Collagen was reported to be active in numerous biological processes in cancers [12]. Collagen type XI alpha 1 chain (COL11A1), a subtype of fibrillary type collagen, plays a role in extracellular matrix and tissues and was over-expressed in human tumors [13]. COL11A1 has also been regarded as a promising prognostic biomarker for breast cancer [13]. On the basis of existing literature data, we carried out studies in an effort to investigate the possible CDX2 may act as a significant cytokine in breast cancer. This study distinguishes from other studies by emphasizing the role of transcriptional factors and miRNAs in the invasion, migration, and metastasis of breast cancer epithelial cells.

## Methods and materials

### Ethical statement

All patients participated in this study signed informed consent and the study protocols were approved by the Ethics Committees of Harbin Medical University Cancer Hospital. All animal experiments in this study have been approved by the Ethics Committee of Harbin Medical University Cancer Hospital. The animals received humane care according to the Guide for the Care and Use of Laboratory Animals published by the US National Institutes of Health.

### Breast cancer tissue samples

Breast cancer tissue samples were collected from 86 patients (49 cases aged < 50 years old; 37 cases aged ≥ 50 years old) undergoing surgical resection of breast cancer at the Harbin Medical University Cancer Hospital between April 2014 and August 2017. In addition, adjacent normal tissue samples were also resected from all patients as controls. The samples used were not subjected to preoperative radiotherapy and/or chemotherapy, and were diagnosed as non-specific invasive breast cancer by more than two associate chief physicians at the Department of Pathology. After diagnoses, the samples were classified according to World Health Organization tumor classification standard. There were 51 samples classified as grade I + II (benign and atypical), and 35 samples classified as grade III (anaplastic). Next, the tumors were evaluated based on the sixth edition of the American Joint Committee on Cancer tumor, node, metastases staging system (stage I/II, n = 57; stage III, n = 29). No lymphatic metastasis was found in the samples, which were fixed with 10% formalin, dehydrated, paraffin-embedded and sectioned into 4 μm for further experimentation.

### Immunohistochemistry

Paraffin-sectioned tissues sections were dewaxed and dehydrated. The target antigens were retrieved by microwaving the sections in Tris-ethylenediaminetetraacetic acid (EDTA) buffer containing 10 mM Tris and 1 mM EDTA (pH = 9.0) The samples were heated in a microwave at 800 W for 3 min and then for 7 min at 400 W to continue boiling. The sections were cooled down for 30 min at room temperature. After that, the 10-min heating procedure was repeated. After being blocked with normal goat serum (C-0005, Shanghai Haoraobio Science & Technology Co., Ltd., Shanghai, China), the tissues were incubated with the primary rabbit anti-human antibody to CDX2 (dilution ratio of 1:100, ab88129) and secondary goat anti-rabbit immunoglobulin G (IgG; dilution ratio of 1:1000, ab6785) at 37 °C for 20 min, followed by incubation with horseradish peroxidase (HRP)-conjugated Streptavidin (0343-10000U, Imunbio Co., Ltd, Beijing, China) at 37 °C for 20 min. Next, the tissues were developed using diaminobenzidine (ST033, Whiga Biosmart Co., Ltd., Guangzhou, Guangdong, China), counterstained with hematoxylin (PT001, Shanghai Bogoo Biotechnological Co., Ltd., Shanghai, China) for 1 min, and returned to blue using 1% ammonium hydroxide, mounted and observed by microscopy. Five high power field of vision were randomly selected from each slice with 100 cells in per field of vision. Sections with less than 10% positive-cells were regarded as negatively expressed, more than 10% and less than 50% were considered as positively expressed, and more than 50% were considered as strongly positively expressed.

### Cell culture and treatment

Normal human breast epithelial cell line HBL-100, and human breast cancer epithelial cell lines MCF-7 and MDA-MB-231 were procured from the Cell Bank of Chinese Academy of Sciences (Shanghai, China), and the cell line exhibiting the lowest expression of CDX2 was selected using RT-qPCR.

Subsequently, plasmids of over-expressed CDX2, let-7b mimic, let-7b inhibitor, COL11A1 over-expression, shRNA targeting COL11A1 and their relevant negative control23 (NC), and lentiviral expression vectors of pGC-FU-GFP and pLVX-shRNA were constructed by Sangon Biotech (Shanghai, China). Cells at passage three were transfected with the above-mentioned plasmids using Lipofectamine 2000 (Invitrogen, Carlsbad, CA, USA). Next, the stably transfected cells were screened and cultured in the medium with G418 (1000–2000 μg/mL) for 4 weeks.

### RNA isolation, reverse transcription, and quantitative real-time polymerase chain reaction

Total RNA content was extracted from breast cancer and adjacent normal tissues using the TRIZOL reagent (15596-018, Beijing Solarbio Science & Technology Co., Ltd., Beijing, China) and then reverse transcribed into complementary DNA (cDNA) in strict accordance with the manufacturer’s protocols of the kit (K1622, Beijing Reanta Biotechnological Co., Ltd, Beijing, China). Primers were synthesized by Takara Bio (Dalian, Liaoning, China) (Table 1). Glyceraldehyde-3-phosphate dehydrogenase (GAPDH) was regarded as the internal reference primer and the relative transcriptional levels of the target genes were calculated using the 2^−ΔΔCt^ method.

**Table 1:** Primer sequences for RT-qPCR

Genes	Forward primer sequences (5′-3′)	Reverse primer sequences (5′-3′)
CDX2	CCACGCTGGGGCTCTCT	GTCTAGCAGAGTCCACGCTC
let-7b	TGAGGTAGTAGGTTGTGTGGTT	GCGAGCACAGAATTAATACGAC
GAPDH	ACCTGACCTGCCGTCTAGAA	TCCACCACCCTGTTGCTGTA
COL11A1	TGGTGATCAGAATCAGAAGTTCG	AGGAGAGTTGAGAATTGGGAATC

CDX2, caudal type homeobox 2; let-7b, microRNA let-7b; COL11A1, collagen type XI alpha 1 chain; GAPDH, glyceraldehyde-3-phosphate dehydrogenase

### Western blot

After isolation from tissues and cell, protein was then quantified with bicinchoninic acid assay protein assay kit (20201ES76, Yeasen Biotechnology Co., Ltd., Shanghai, China). After quantification, the proteins were separated using polyacrylamide gel electrophoresis and transferred onto a polyvinylidene fluoride membrane. Subsequently, the membrane was hybridized with primary rabbit anti-human antibodies to CDX2 (dilution ratio of 1:2000, ab88129), COL11A1 (dilution ratio of 1:1000, ab64883), E-cadherin (dilution ratio of 1:500, ab15148), Vimentin (dilution ratio of 1:2500, ab92547) and GAPDH (dilution ratio of 1:2500, ab9485) and with the goat anti-rabbit IgG labeled by HRP (dilution ratio of 1:20,000, ab205718). All aforementioned antibodies were purchased from Abcam Inc. (Cambridge, UK). After the membrane was developed, protein quantitative analysis was performed using the ImageJ 1.48u software (National Institutes of Health, Bethesda, MA, USA).

### Chromatin immunoprecipitation (ChIP)

MCF-7 cells were fixed with formalin for 10 min to allow crosslinking of DNA and proteins. The ultrasonic breaker was set to 10 s per ultrasonic cycle with 10-s intervals with 15 cycles to break the chromatin. Then, the supernatant was harvested and divided into two tubes, one being supplemented with IgG (ab6785, Abcam) and the other added with target protein-specific antibodies to CDX2 (sc-134362, 2 µG/1 mL cell lysate, Santa Cruz Biotechnology Inc, Santa Cruz, CA, USA) respectively. Next, the DNA–protein complex was precipitated using Protein Agarose/Sepharose, followed by de-crosslinking. DNA fragments were extracted and purified using a phenol/chloroform mixture. Finally, the binding of let-7b promoter region was detected using specific primers of the let-7b promoter.

### Dual-luciferase reporter gene assay

According to the sequence of let-7b mRNA promoter region binding to CDX2, let-7b promoter wild type (WT) sequence and mutation (Mut) sequence were designed. Simultaneously, MCF-7 cells were plated on a 24-well plate. The luciferase reporter plasmids COL11A1-WT-Luc and COL11A1-Mut-Luc were co-transfected with let-7b mimic or NC of let-7b mimic into the MCF-7 cells upon reaching 80% confluence. At 48 h after transfection of the reporter plasmid, the cells were harvested and reporter assays were performed using a dual luciferase reporter assay system (D0010, Beijing Solarbio Science & Technology Co., Ltd, Beijing, China). The fluorescence intensity was determined using a GLomax20/20 Luminometer (Promega Corporation, Madison, WI, USA).

### 5-ethynyl-2′-deoxyuridine (EdU) staining

MCF-7 cells seeded in 96-well plates at a density of 5 × 10^4^ cells/well were transfected with miRNA as described. Proliferation assays were performed 48 h after transfection in accordance with the manufacturer’s protocol. In brief, after incubation with medium containing EdU for 2 h, the cells were detached, fixed with 4% paraformaldehyde for 30 min and incubated with reagents of B, C, D, and E based on the EdU kit protocols (Guangzhou RiboBio Co., Ltd., Guangzhou, Guangdong, China). The cells were finally incubated with Hoechst33342 staining solution and then photographed using fluorescence microscopy. Cells presenting with red-stained nucleus were regarded as positive cells. EdU labeling rate calculations were performed as previously described [7].

### Transwell assay

MCF-7 cells were resuspended and seeded evenly in a 24-well plate (density of 3 × 10^4^ cells/well), with 200 µL culture medium added to the apical chamber, and complete media in the basolateral chamber wells serving as a chemo-attractant. Subsequently, the plates were incubated with CO_2_. At 24 h, cells on the upper surface of the membrane were removed using a cotton swab, and the basolateral chamber was fixed with 800 μL mixture of methanol-glacial acetic acid at a ratio of 3:1 for 20 min. Transwell chambers were then stained with 500 μL crystal violet for 5 min. The stained cells were photographed and counted under an optical microscope. Subsequently, the average number of cells was calculated and recorded.

Matrigel stored at − 20 °C was melted and diluted with pre-cooled serum-free medium at a ratio of 1:9. Next, Transwell apical chamber was seeded in a 24-well plate and coated with a total of 50 L diluted Matrigel. Transwell invasion assay was carried out following a similar procedure as the Transwell migration assay. The number of cells that invaded the membrane was measured and recorded.

### Tumor xenografts in nude mice

BALB/c nude mice (aged 5 to 7 weeks; weighing 19–21 g) were purchased from Shanghai Lingchang Biological Technology Co., Ltd. (Shanghai, China) and raised under specific-pathogen-free conditions. The nude mice were then assigned into different groups with 8 mice per group and inoculated with transfected MCF-7 cells.

The cells were dispersed into a cell suspension (5 × 10^7^ cells/mL). Nude mice were subcutaneously injected with 0.2 mL cell suspension in the left armpits. Then, tumor growth was recorded on the 7th, 14th, 21st and 28th day after injection using Vernier calipers. Tumor volume was calculated 3 times with the formula π (2ab)/6, in which a referred to the short diameter, while b was the long diameter. The mice were euthanized on the 28th day and tumors were extracted. Tumor tissues were then fixed with 10% formalin, dehydrated, paraffin-embedded and serially cut into 4-µm thick sections for RNA and protein content extraction. Meanwhile, hematoxylin and eosin (H&E) staining of the lungs and livers was performed in order to observe lymph node metastasis.

### Statistical analyses

All measurement data were shown as mean ± standard deviation and analyzed using the SPSS 21.0 software (IBM Corp., Armonk, NY, USA). Comparisons of statistics with normal distribution and uniformed variance between two groups were analyzed using the paired *t* test or unpaired *t*-test. One-way analysis of variance (ANOVA) was performed for comparisons among groups followed by Tukey’s post hoc test. Repeated measures ANOVA was carried out for comparisons among groups at different time points followed by Bonferroni post hoc test. A value of *p* < 0.05 was considered to be statistically significant.

## Results

### CDX2 is poorly expressed in breast cancer, while over-expressed CDX2 inhibits migration and invasion of breast cancer epithelial cells

**Fig. 1:**
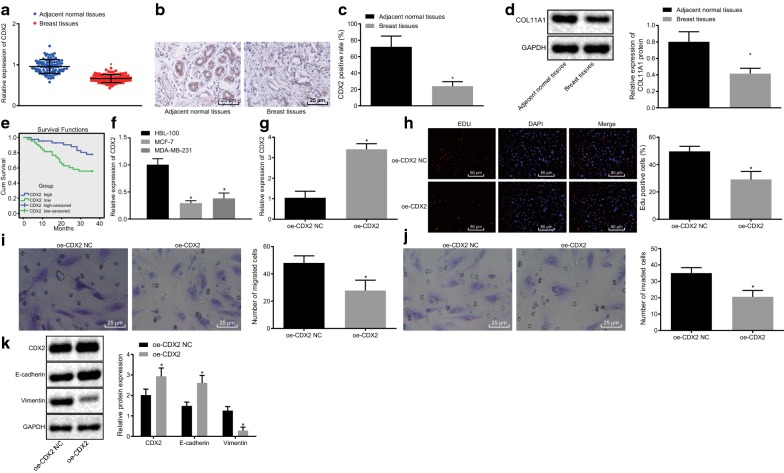
CDX2 is poorly expressed in breast cancer, and overexpression of CDX2 suppresses migration and invasion of breast cancer epithelial cells. **a** CDX2 mRNA expression detected by RT-qPCR (n = 86); **b** immunohistochemical images of CDX2 in breast cancer and adjacent normal tissues (×400); **c** CDX2 positive rate in tissues measured by Immunohistochemistry (n = 86); **d** relative expression of CDX2 protein in tissues examined by western blot analysis (n = 86); *p < 0.05 vs. adjacent normal tissues; **e** CDX2 in breast cancer survival rate analyzed by Kaplan–Meier; **f** breast cancer cell line with the lowest expression of CDX2 screened by RT-qPCR; *p < 0.05 vs. normal human breast epithelial cell line HBL-100; **g** CDX2 mRNA expression in MCF-7 cells detected by RT-qPCR after 48-h of transfection; **h** MCF-7 cell proliferation ability determined by EdU assay after 48-h of transfection (×200); **i** MCF-7 cell migration ability examined by Transwell migration assay (×200); **j** MCF-7 cell invasion ability (×200); **k** CDX2, E-cadherin and Vimentin protein expression in MCF-cells after 48-h of transfection; *p < 0.05 vs. oe-CDX2 NC. Measurement data were described as mean ± standard deviation. Paired *t*-test was applied for **a**, **c**, and **d**, while unpaired *t*-test was conducted for **f**−**k** analyses. Each cellular experiment was repeated 3 times

As compared to adjacent normal tissues, CDX2 expression was decreased in breast cancer tissues, and the CDX2 protein was located at the nucleus and stained in brown (*p* < 0.05; Fig. 1a–c). Western blot analysis demonstrated that the CDX2 protein was poorly expressed in breast cancer tissues (Fig. 1d). Analysis of survival rate revealed that down-regulation of CDX2 was evident in patients with low survival rates (Fig. 1e).

Furthermore, the expression of CDX2 was diminished in human breast cancer epithelial cell lines MCF-7 and MDA-MB-231 compared to that of normal human breast epithelial cell line HBL-100, where the MCF-7 cell line exhibited the lowest expression of CDX2 (*p* < 0.05; Fig. 1f). Thereby, MCF-7 cells were chosen for further experimentation.

Subsequently, Fig. 1g demonstrated that oe-CDX2 was successfully delivered, and further experiments were continued. In addition, results from EdU assay revealed that EdU positive-cells treated with over-expressed CDX2 showed a marked decrease compared to the positive-cells in NC of over-expressed CDX2 (*p* < 0.05; Fig. 1h). Moreover, over-expression of CDX2 inhibited the migration and invasion of MCF-7 cells (*p* < 0.05; Fig. 1i, j). Western blot results further demonstrated that CDX2 and E-cadherin protein expression was enhanced in cells treated with over-expressed CDX2, while the protein expression of Vimentin showed a significant decline (*p* < 0.05; Fig. 1k) compared to the expression in NC of over-expressed CDX2. These results collaboratively illustrated that over-expressed CDX2 inhibited the proliferation, migration, and invasion abilities of breast cancer epithelial cells.

### CDX2 inhibits migration and invasion of breast cancer epithelial cells by up-regulating let-7b

**Fig. 2:**
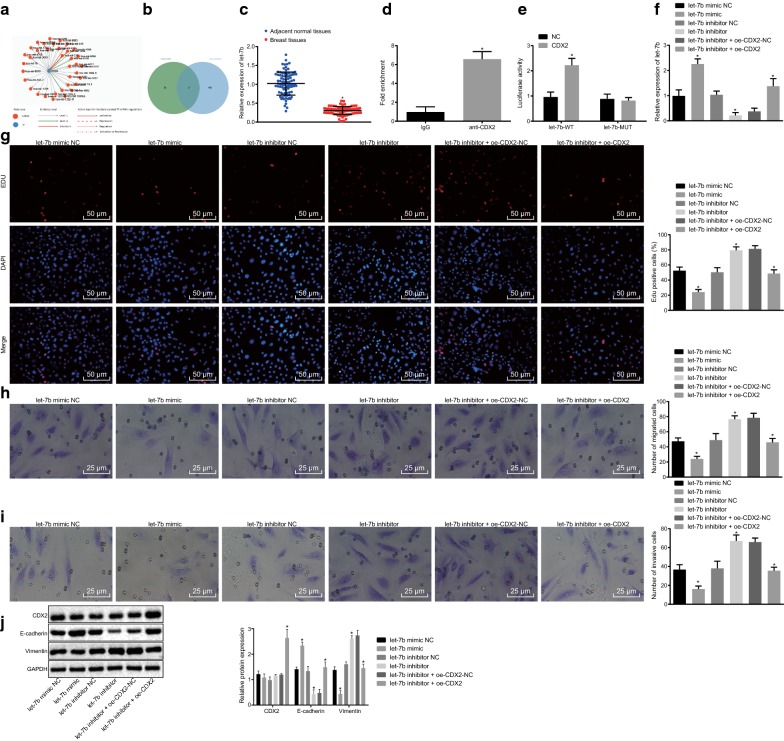
CDX2 over-expression up-regulates let-7b to suppress migration and invasion of breast cancer epithelial cells. **a** CDX2-mediated miRNAs predicted by TransmiR database; **b** comparison between CDX2-regulated miRNAs predicted by TransmiR database and poorly expressed miRNAs in breast cancer-relevant microarray GSE456666; **c** the expression of let-7b in tissues detected by RT-qPCR (n = 86); **d** binding relation between let-7b promoter region and CDX2 detected by ChIP; *p < 0.05 vs. IgG; **e** Luciferase activities of CDX2 and let-7b; *p < 0.05 vs. NC; **f** let-7b expression in MCF-7 cells with different treatments after 48-h of transfection; **g** MCF-7 cells proliferation ability determined by EdU assay after 48-h of transfection (×200); **h** MCF-7 cells migration ability examined by Transwell migration assay (×200); **i** MCF-7 cells invasion ability examined by Transwell invasion assay (×200); **j** CDX2, E-cadherin and Vimentin protein expression in MCF-7 cells with different treatments tested by western blot analysis after 48-h of transfection. *p < 0.05 vs. NC. The aforementioned data were measurement data and expressed as mean ± standard deviation. Paired *t*-test was carried out for analysis in **c**; differences in **d**–**j** were analyzed with unpaired *t*-test. The experiment was repeated 3 times

TransmiR database and the relevant microarray of breast cancer, GSE45666 (Fig. 2a, b) indicated that CDX2 may mediate the let-7b. Subsequent RT-qPCR displayed that the expression of let-7b was decreased in breast cancer tissues in contrast to adjacent normal tissues (*p* < 0.05; Fig. 2c). The CDX2 promoter region in let-7b was identified using ChIP assay, and results confirmed that CDX2 was enriched in let-7b (Fig. 2d). Meanwhile, dual-luciferase reporter gene assay results demonstrated that the luciferase activity of cells transfected by over-expressed CDX2 and let-7b WT was visibly enhanced when compared with NC (*p* < 0.05). However, no significant differences were detected in luciferase intensity in the Mut plasmid (*p* > 0.05; Fig. 2e).

Subsequently, RT-qPCR was carried out to evaluate the efficiency of transfection, which substantiated that the delivery was successful (*p *<0.05). After co-transfection with CDX2 over-expressed plasmid, let-7b mRNA was found to be highly expressed in cells treated with inhibited let-7b and over-expressed CDX2 when compared with the relevant NC (*p* < 0.05; Fig. 2f).

Furthermore, results obtained from EdU, Transwell and western blot analysis (Fig. 2g–j) revealed that EdU positive-cells were decreased, cell migration and invasion abilities were suppressed, E-cadherin proteins were increased while Vimentin protein expression was significantly reduced in let-7b mimic-treated MCF-7 cells when compared with its NC (*p* < 0.05). However, let-7b mimic had no impact on the protein expression of CDX2 (*p* > 0.05). The results were reciprocal in the comparisons between the let-7b inhibitor and its NC with unchanged protein expression of CDX2 (*p* > 0.05). Meanwhile, in cells treated with both let-7b inhibitor and CDX2 over-expression, EdU positive-cells were reduced, cancer cells migration and invasion were restrained, E-cadherin and CDX2 protein expression was increased and Vimentin protein expression was decreased relative to NC of let-7b inhibitor and over-expressed CDX2 (*p* < 0.05). These data showed that over-expression of CDX2 up-regulated the expression of let-7b, consequently inhibiting the migration and invasion of breast cancer epithelial cells.

### Let-7b reduces breast cancer cell migration and invasion by directly down-regulating COL11A1

**Fig. 3:**
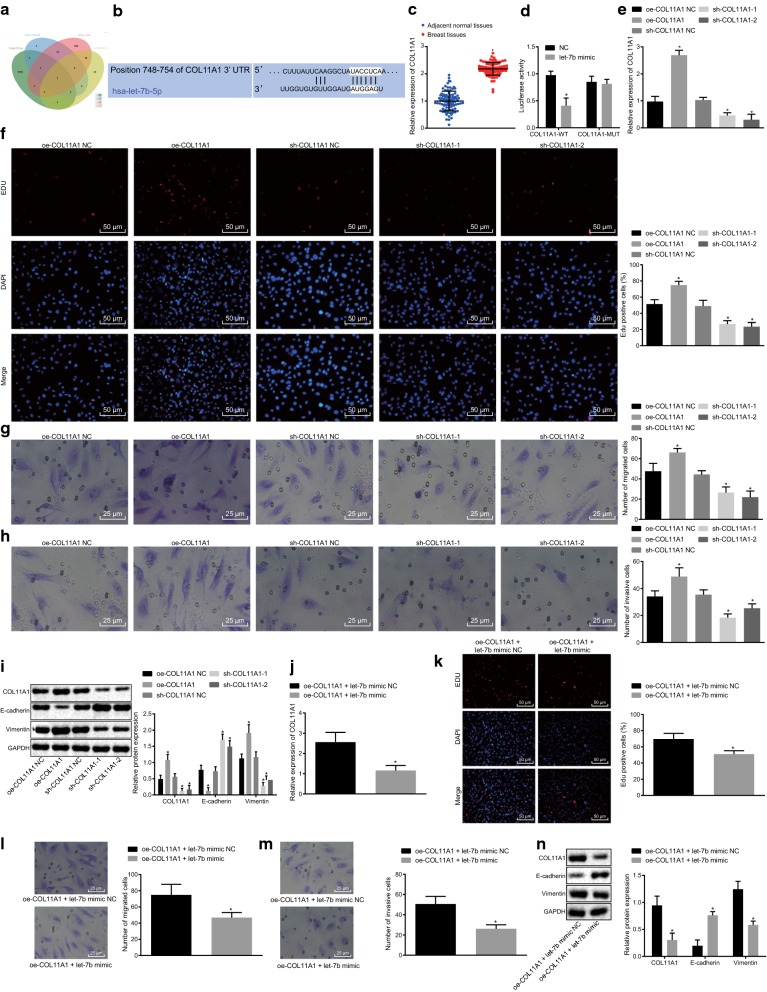
Let-7b reverses the effect of COL11A1 over-expression on breast cancer cell migration and invasion. **a** let-7b target genes predicted by TargetScan database and highly expressed genes in microarrays GSE109169, GSE3744 and GSE26910; **b** binding sites of let-7b and COL11A1; **c** COL11A1 mRNA expression in clinical tissues detected by RT-qPCR (n = 86); **d** Luciferase activity of let-7b and COL11A1; *p < 0.05 vs. NC; **e** COL11A1 mRNA expression in MCF-7 cells treated with depleted COL11A1 determined by RT-qPCR after 48-h of transfection; **f** proliferation of MCF-7 cells treated with silenced COL11A1 expression detected by EdU assay after 48-h of transfection (×200); **g** MCF-7 cell migration ability after COL11A1 knockdown determined by Transwell migration assay (×200); **h** MCF-7 cell invasion ability after COL11A1 knockdown examined by Transwell invasion assay (×200); **i** CDX2, E-cadherin and Vimentin protein expression in MCF-7 cells treated with silenced COL11A1 detected by western blot analysis after 48-h of transfection; **j** COL11A1 mRNA expression in MCF-7 cells with different treatments determined by RT-qPCR after 48-h of transfection; **k** MCF-7 cells proliferation tested by EdU assay after 48-h of transfection (×200); **l** MCF-7 cell migration ability (×200); **m** MCF-7 cells invasion ability (×200); **n** CDX2, E-cadherin and Vimentin protein expression in MCF-7 cells detected by western blot analysis after 48-h of transfection. *p < 0.05 vs. NC. The above statistics were measurement data, which were expressed as mean ± standard deviation. Except for **c** which was analyzed by paired *t*-test, analyses in **d**–**n** were carried out using unpaired *t*-test. Each cellular experiment was performed at least 3 times

In order to realize the target genes of let-7b, highly-expressed genes in breast cancer were predicted using the TargetScan database (Fig. 3a) and breast cancer-related microarrays including GSE109169, GSE3744, and GSE26910, which suggested that the COL11A1 gene may be a target of let-7b. Meanwhile, the binding sites between let-7b and COL11A1 were predicted using the TargetScanHuman website (http://www.targetscan.org/; Fig. 3b). RT-qPCR demonstrated that COL11A1 was highly expressed in cancer tissues compared to adjacent normal tissues (*p* < 0.05; Fig. 3c). The results of dual-luciferase reporter gene assay revealed that the luciferase activity in co-transfection of let-7b mimic and COL11A1 WT was remarkably decreased when compared with its NC (*p* < 0.05), while the luciferase activity in Mut plasmid displayed no obvious differences (*p* > 0.05; Fig. 3d).

Next, RT-qPCR was conducted to assess the over-expression or silencing efficiency of delivery (Fig. 3e). Results demonstrated that COL11A1 expression was increased when the cells were treated with over-expressed COL11A1 compared with its NC (*p* < 0.05), while COL11A1 expression was declined in cells treated with sh-COL11A1 in contrast to that in NC of sh-COL11A1 (*p* < 0.05). Meanwhile, the results obtained from EdU, Transwell and western blot analysis (Fig. 3f–i) revealed that EdU positive-cells were increased, migration and invasion were promoted, COL11A1 and Vimentin protein expression was enhanced and the protein expression of E-cadherin was decreased in MCF-7 cells treated with over-expressed COL11A1 compared with that in NC of over-expressed COL11A1. MCF-7 cells treated with sh-COL11A1 showed reduced EdU positive-cells, migration and invasion as well as COL11A1 and Vimentin protein expression, while enhanced E-cadherin protein expression relative to sh-COL11A1 NC (*p* < 0.05).

Furthermore, cells with over-expressed COL11A1 were co-transfected with let-7b mimic plasmid to investigate the regulatory mechanism between let-7b and COL11A1. Results from RT-qPCR illustrated that the expression of COL11A1 was diminished after co-transfection with over-expressed COL11A1 and let-7b mimic compared to its NC (Fig. 3j). Results from EdU, Transwell and western blot analysis (Fig. 3k–n) revealed that when MCF-7 cells were treated with over-expressed COL11A1 and let-7b mimic, EdU positive-cells, cells migration and invasion abilities as well as the protein expression of COL11A1 and Vimentin were down-regulated, while the protein expression of E-cadherin was increased when compared to the NC of over-expressed COL11A1 and let-7b mimic (*p* < 0.05). These data strongly indicated that let-7b may downregulate breast cancer cell migration and invasion via direct repression of COL11A1.

### CDX2/let-7b/COL11A1 modulates growth and metastasis of breast cancer in vivo

**Fig. 4:**
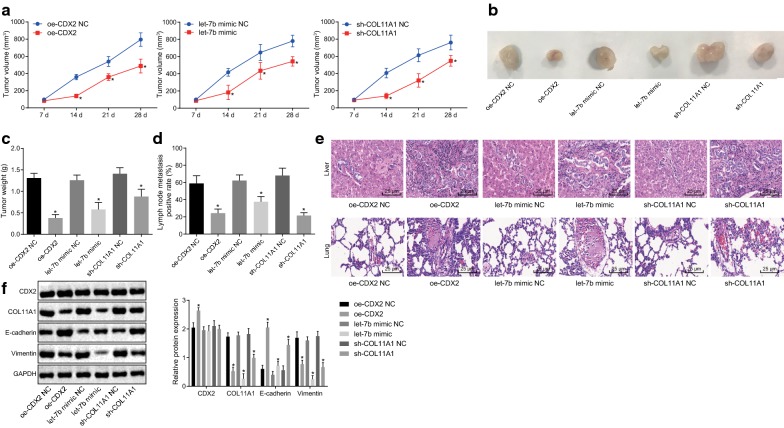
CDX2 or let-7b over-expression and COL11A1 silencing inhibits breast cancer growth and metastasis. **a** Tumor growth curve of nude mice transfected with over-expressed CDX2, let-7b mimic and shRNA against COL11A1 on the 7th, 14th, 21st and 28th day after injection; On the 28th day, the nude mice in each group were euthanized, and the tumors were taken. **b** Representative images of xenograft tumors in nude mice with various transfections; **c** average weight of xenograft tumors; **d** lymph node metastasis positive rate in different operations; **e** representative images of HE staining of metastatic nodules in the livers (upper) and lungs (lower) (×400); **f** relative protein expression of CDX2, COL11A1, E-cadherin and Vimentin determined by western blot analysis. *p < 0.05 vs. NC of CDX2 over-expression, NC of shRNA targeting COL11A1 or NC of let-7b mimic (n = 8). All the data in this experiment were measurement data and described as mean ± standard deviation. Unpaired *t*-test was applied for analyses of **c**, **d** and **f**. In **b** repeated ‘measures ANOVA was conducted and followed by Bonferroni post hoc test

On the one hand, MCF-7 cells were subcutaneously injected into the left armpits of nude mice, which were randomized into six groups. We found that both over-expression of CDX2 or let-7b, or down-regulation of COL11A1 efficiently reduced the volume and weight of the tumor, as shown in Fig. 4a–c (*p* < 0.05).

Additionally, lung and liver tissues were extracted from nude mice and made into paraffin samples for lymph node metastasis analysis. H&E staining was employed to examine lymph node metastasis and results demonstrated that the irregular cancer cells of lung and liver tissues and other poorly differentiated characteristics were observed (Fig. 4e). Compared to the NC of CDX2 over-expression vector, positive lymph node metastasis was observed in 2 out of 8 nude mice inoculated with over-expressed CDX2 (positive metastasis rate = 24.35%). H&E staining further showed that the tumor cells were decreased in response to treatment with CDX2 overexpression (*p* < 0.05). In contrast to NC of let-7b mimic, positive lymph node metastasis was observed in 3 out of 8 nude mice (with let-7b treatment) where the rate of positive metastasis was 37.5%, and tumor cell number was also evidently reduced (*p* < 0.05). Tumor cells were likewise decreased in nude mice inoculated with shRNA against COL11A1, where the positive metastasis rate was 25% as 2 out of 8 nude mice presented positive lymph node metastasis compared to NC of shRNA against COL11A1 (Fig. 4d).

Western blot analysis (Fig. 4f) demonstrated that after treatment with over-expressed CDX2, the expression of E-cadherin was increased, while the expression of COL11A1 and Vimentin was decreased compared with its relevant NC (*p* < 0.05). Similarly, E-cadherin expression was also enhanced while the expression of COL11A1 and Vimentin was reduced upon treatment with let-7b mimic in contrast to that of its NC (*p* < 0.05). When treated with shRNA targeting COL11A1, CDX2 showed no significant difference (*p* > 0.05), the expression of E-cadherin was elevated, while the expression of COL11A1 and Vimentin was reduced compared with NC of shRNA against COL11A1 (*p* < 0.05). These data indicated that let-7b or CDX2 up-regulation or COL11A1 down-regulation can inhibit breast cancer growth and metastasis.

## Discussion

Tumor cells metastases led by tumor cell invasion and migration are regarded as the most fatal stage in cancers. For example, breast cancer metastases can be widely disseminated to several regions in the human body including the brain, bone, liver, and lung [14]. Furthermore, metastasis often occurred before diagnosis in breast cancer [15]. Therefore, it is trivial to seek effective prognostic markers to predict metastasis in breast cancer. Burgeoning genetic researches have been widely reported on tumor cells invasion, migration and metastasis. A previous study highlighted the importance of genetic factors elucidation in the pathogenesis of breast cancer [16]. However, limited studies have explored the gene regulatory networks implicated in breast cancer. In the current study, we proposed that breast cancer metastasis was correlated with CDX2, and CDX2 could inhibit breast cancer progression by up-regulating let-7b.

Initially, we found that CDX2, which was frequently studied in intestinal cancer, was associated with the survival of patients with breast cancer. Our findings further indicated that CDX2 was poorly expressed in breast cancer, while the over-expressed CDX2 diminished the migration and invasion abilities of breast cancer epithelial cells. As a homeotic transcriptional factor, CDX2 was poorly expressed in colon cancer and associated with invasion and metastasis of cells [17]. Therefore, it would be reasonable to suggest that poor expression of CDX2 is correlated with migration and invasion of breast cancer epithelial cells, and could be countered by over-expression of CDX2.

A previous study has demonstrated that miRNAs were regulated by CDX2 [18]. Similarly, we uncovered that CDX2 inhibited the migration and invasion of breast cancer epithelial cells by up-regulating let-7b. Let-7b has also been previously highlighted as a tumor suppressor due to its involvement in various mechanisms, including cell proliferation and invasion [19]. Similarly, another study displayed that let-7b was poorly expressed in patients diagnosed with breast cancer at clinical stages I–IV [20]. In addition, our data demonstrated that let-7b mimic transfection resulted in the inhibition of cells invasion and migration, while poor prognoses were occurred in breast cancer patients with reduced let-7b expression, suggesting that let-7b may be a potential biomarker for breast cancer cell behavior. With regard to regulation of miRNAs in cancers, accumulating evidence put emphasis on the specific miRNA-regulated mechanisms and indicated that let-7b could serve as an effective index in distinguishing breast cancer clinical behaviors [21]. Furthermore, He et al. found that CDX2 bound to the promoter region and inhibited miR-145-5p transcription, thus relieving the suppressive effect of miR-145-5p on the translation of small ubiquitin-like modifier protein-specific protease 1 and affecting the invasion and migration of prostate cancer cells [22]. In the present study, we first predicted the targeting relationship between CDX2 and let-7b using the TargetScan website with breast cancer microarray data, and examined the binding of CDX2 to the let-7b promoter region with ChIP assay. Based on these experiments, it was confirmed that CDX2 targets let-7b. These findings highlight the gene regulatory network, where genes and miRNAs were intertwined in breast cancer epithelial cells activities.

Furthermore, we explored the target genes of let-7b in the current study. The COL11A1 gene was screened using the TargetScan database, and subsequently, the binding sites between let-7b and COL11A1 were predicted by the TargetScanHuman website. The targeting relationship was substantiated by a dual luciferase reporter gene assay. Meanwhile, a prior research reported that COL11A1 was highly expressed in cancers and potentiated the invasion by coordinating with invasion-sensing biomarkers. However, there were no reports on the direct association between COL11A1 and breast cancer in the study [23]. Another study unraveled that COL11A1 expression was found in fibroblasts associated with breast cancer and played a vital role in the metastasis of tumor cells [24]. In the present study, over-expressed CDX2 and let-7b inhibited lymph node metastasis and tumor growth by silencing COL11A1.

## Conclusion

**Fig. 5:**
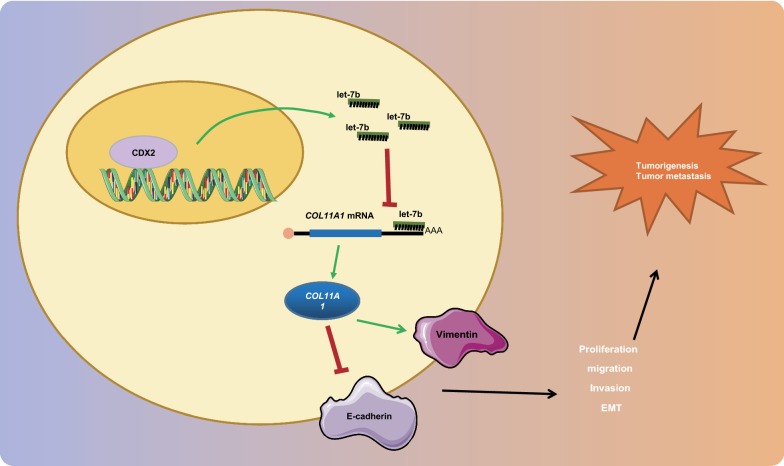
CDX2 promotes the expression of let-7b and inhibits COL11A1, thus suppressing the proliferation, invasion, and migration abilities of breast cancer cells

Overall, the current study verified the CDX2/let-7b/COL11A1 modulation in breast cancer epithelial cells, and investigated the tumor growth and metastasis through in vivo models (Fig. 5). Our findings highlight the role of gene regulatory networks in tumor cell metastasis, which unlocks novel avenues for breast cancer therapy. However, more in-depth investigations and optimization are needed in future studies to ensure the successful therapeutic biomarker discovery for the treatment and prognosis of breast cancer.

## Data Availability

The datasets generated/analyzed during the current study are available.

## Abbreviations

CDX2: caudal type homeobox 2
COL11A1: collagen type XI alpha 1 chain
IHC: immunohistochemistry
NC: negative control
cDNA: complementary DNA
GAPDH: glyceraldehyde-3-phosphate dehydrogenase
ChIP: chromatin immunoprecipitation
WT: wild type
Mut: sequence and mutation
EdU: 5-ethynyl-2′-deoxyuridine
ANOVA: analysis of variance
